# QTL Analysis of Shading Sensitive Related Traits in Maize under Two Shading Treatments

**DOI:** 10.1371/journal.pone.0038696

**Published:** 2012-06-19

**Authors:** Liuzheng Yuan, Jihua Tang, Xiuping Wang, Chaohai Li

**Affiliations:** College of Agronomy, Henan Agricultural University, Zhengzhou, China; Pennsylvania State University, United States of America

## Abstract

During maize development and reproduction, shading stress is an important abiotic factor influencing grain yield. To elucidate the genetic basis of shading stress in maize, an F_2:3_ population derived from two inbred lines, Zhong72 and 502, was used to evaluate the performance of six traits under shading treatment and full-light treatment at two locations. The results showed that shading treatment significantly decreased plant height and ear height, reduced stem diameter, delayed day-to-tassel (DTT) and day-to-silk (DTS), and increased anthesis-silking interval (ASI). Forty-three different QTLs were identified for the six measured traits under shading and full light treatment at two locations, including seven QTL for plant height, nine QTL for ear height, six QTL for stem diameter, seven QTL for day-to-tassel, six QTL for day-to-silk, and eight QTL for ASI. Interestingly, three QTLs, *qPH4*, *qEH4a*, and *qDTT1b* were detected under full sunlight and shading treatment at two locations simultaneously, these QTL could be used for selecting elite hybrids with high tolerance to shading and high plant density. And the two QTL, *qPH10* and *qDTS1a*, were only detected under shading treatment at two locations, should be quit for selecting insensitive inbred line in maize breeding procedure by using MAS method.

## Introduction

Maize (*Zea mays* L.) is a crop derived from the (sub) tropics, and has been imported and cultivated in many areas of higher geographic latitude around the world. In the temperate regions, cultured maize hybrids have been faced many abiotic stresses in the field, such as drought, high or low temperature, and cloudy and rainy climate. Among them, persistent shading has become a restrictive meteorological factor that affects normal plant development and reduces grain yield, especially when accompanied by increasing plant density in many area of the world. Reed et al. reported that when plants were shaded during flowering, photosynthesis decreased and kernel abortion increased [1]. In addition, when plants were shaded during grain fill, kernel weight and yield were reduced; thus, kernel number and grain yield can be increased or decreased by either increased light or shading plants during the reproductive period, respectively [2]–[5]. Work by Tollenaar and Daynard [6], and Gerakis and Papakosta-Tasopoulou [7] has also shown the feasibility of using shading stress to affect grain yield in maize. Additionally, shading in maize during different developmental stages not only decreases grain yield, but also affects the normal development of other agricultural traits, such as internode length reduction [8], delayed flowering and silking time [9], decreased kernel set in the apical ear region or varying degrees of barrenness [10]–[11], inhibiting silk elongation [12], increased or decreased plant height, delayed new leaves appearance [9], and reduced leaf thickness [13].

Many researchers have emphasized the variation in grain yield and several agricultural traits, as well as phytohormone content, during different shading treatments [14]–[16]. In the studies on the genetic variation of the effect of shade treatment on grain yield and several agricultural traits in maize, Early et al. showed that tolerance to shade of several hybrids was significantly different [17]. In addition, shading during the vegetative phase and the reproductive phase was more detrimental to two hybrids with respect to number of kernels and grain yield [2]. Hébert et al. found that biomass allocation was significantly affected by light treatments, and that the effects varied among genotypes and showed significant interactions between genotype and shading [18]. Liu and Tollenaar reported that heterosis for grain yield was greater when plants were exposed to shading during the presilking and silking periods compared to the unshaded control [19].

Many types of abiotic stress, such as water deficit and abnormal temperature, can cause phenotypic variation in plant height, ear height, flowering and silking time, anthesis-silking interval (ASI). Many reports, therefore, have emphasized dissecting the genetic basis of these agricultural traits using QTL mapping methods under different abiotic stress, and many QTLs have been identified for these traits [20]–[26]. Although shading is an important abiotic stress factor that affects morphological and flowering-related traits as well as grain yield, there are few reports of the genetic basis of these important traits under shading treatment.

In many parts of the world, during the maize growth season, it always is overcast and rainy, and the low light level is an important stress factor that affects maize normal development and grain yield, especially when accompanied by the increasing plant density in many areas. For example, in the Huanghuihai maize belt in China, maize is always planted after wheat is harvested, and its flowering and grain filling stage is always between August to September, which is always rainy. The average reduction of grain yield is 10%–15% because of the low light shading stress, and can reach to 20%–30% reduction in certain years. Shading stress frequently occurs in maize developmental and reproductive stages, resulting in a serious reduction of grain yield. To prevent the effect of shading stress in maize, an effective strategy would be to develop an elite hybrid with high shading stress tolerance. Thus, selection of an elite hybrid with tolerance to shading has become an important target for maize breeders. However, the genetic basis of the effect of shading on the main agricultural traits is still unclear. The objectives of this study were to (i) elucidate the influence of shading treatment for some agricultural traits in the field, (ii) identify QTLs for these traits under shading treatment conditions.

## Materials and Methods

### Experimental Population and Field Treatment

An F_2:3_ population comprising 206 individuals was constructed using two elite inbred lines, Zhong72 and 502. The inbred line Zhong72 has a strong ability to endure shading stress, and is selected from an exotic hybrid including a part of tropical germplasm. The other parent, 502, is sensitive to shading stress, and was selected from a local Chinese germplasm, Tangsipingtou [27].

In 2008, the F_2:3_ population, the two inbred lines and its hybrid were evaluated at the farms of Henan Agricultural University (Zhengzhou, E113°42', N34°48' ) at 28 of April, which is located in northern China has an average yearly temperature of 14.3°C and 640.9 mm of average rainfall per year, and Xunxian Agricultural Institute (Xunxian, E114°33', N35°41'), which is located in the center of Northern China Plain with an average temperature 14.2°C and 784 mm of average rainfall per year, and planted the experimental materials at 14 of June. The experimental materials were evaluated under shading and full-light treatment respectively, and followed a randomized complete block design approach with three replications for each treatment in each location. Each material was sown in one plot consisting of 13 plants in a single 3.5 m long row, with a distance of 0.23 m between two plants. Rows were planted 0.6 m apart, allowing a density of 4850 plants per hectare. To ensure the growth of 13 plants per plot, all plots were over-seeded and only one plant was preserved to reduce competition among seedlings. During the seedling stage, 175 kg N ha^−1^ (urea), 67.5 kg P_2_O_5_ ha^−1^ (calcium superphosphate), and 101.3 kg K_2_O ha^−1^ (potassium nitrate) were applied to the soil, and an additional 175 kg N ha^−1^ (urea) was added before pollination. The full-light treatment (CK) corresponds to the experiment described by Fournier and Andrieu [8], and the shading treatment was identical to that paper, except that plants were planted in an 3.5 m high isolation chamber and shaded from the tip appearance of leaf 8 onwards to 10d after pollen shedding. Shading was accomplished using black polypropylene fabric with 50% light penetration, and the time of shading treatment for the experimental materials was between 7 of June to 15 of July at Zhengzhou, as the time of shading treatment was between 22 of July to August at Xunxian. Field conditions were maintained for maize production. The Climate data were obtained from the Climate Bureau of Zhengzhou, China and Climate Bureau of Xunxian, China, and the base temperature of 10°C was used in this study [28].

### Field Evaluation

Ten plants from the second row of each plot were initially assessed before anthesis in the field, and six traits, including plant height, ear height, stem diameter, day-to-tassel, day-to-silk, and anthesis-silking interval, were evaluated. Day-to-tassel (DTT) was defined as 60% plant tassel sprout out from leaf in one row, day-to-silk (DTS) was defined as 60% plant silk spill out in one row, the anthesis-silking interval (ASI) was calculated as the interval from anthesis to silk. After pollen shedding, the same ten plants were evaluated for plant height (PH), ear height (EH), and stem diameter (SD). Plant height was evaluated from the earth to the top of the tassel; ear height was evaluated from the earth to the node of ear. Stem diameter was evaluated at the diameter of the third internode from earth. The mean value of measured traits for each row was computed, followed by calculation of the measured trait of three replications at full light and shading treatment as well as in both environments. Data analysis was performed using SAS 8.0 statistical software package with the PROC MIXED procedure. The broad-sense heritability (*h* ^2^) of measured traits was computed as previously described by Knapp et al. (1985): *h* ^2^ = σg^2^/(σg^2^+σgl^2^/n+σe^2^/nr), whereσg^2^ is the genetic variance,σgl^2^ is the interaction of genotype with locations,σe^2^ is error variance, r is the number of replications, and n is the number of locations. The estimates of σg^2^, σgl^2^, and σe^2^ were obtained from an analysis of variance (ANOVA) [29].

### Molecular Linkage Construction and QTL Mapping

Polymorphisms between the two parents, Zhong72 and 502, were screened using 560 pairs of simple sequence repeats (SSR) markers selected from the maize genome database (www.maizegdb.org). Two hundred and ten SSR markers possessed distinct polymorphisms in the two parents and were chosen to amplify the F_2_ population DNA. Molecular linkage maps were constructed using Mapmakers 3.0 at a LOD threshold >3.0 [30].

The composite interval mapping method and Model 6 of the Zmapqtl module of QTL Cartographer 2.5 were used to identify QTL using the average data of three replications for each treatment at one location. The statistical model was: *y*_i_ = *b*_0_+*b* ^*^ *x* ^*^_j_+∑*b*_k_ *x*_jk_+*e*_j_ (*k*≠ *i*, _i_+1; for *_j_* = 1, 2, …., n). where *yi*, is the trait value of the *j*th individual, *b*_0_ is the mean of the model, *b* ^*^ is the effect of the putative QTL expressed as a difference in effects between homozygote and heterozygote, *x* ^*^ *_j_* is an indicator variable, taking a value 1 or 0 with probability depending on the genotypes of markers *i* and *j* and the position being tested for the putative QTL, *b_k_* is the partial regression coefficient of the phenotype *y* on the *k*th marker, *x_jk_* is a known coefficient for the *k*th marker in the *j*th individual, taking a value 1 or 0 depending on whether the marker type is homozygote or heterozygote, and *e_j_* is a random variable [31].The threshold of a LOD was calculated using 1000 permutations at a significance level of P = 0.05, with scanning intervals of 2 cM between markers and a putative QTL, and a 10 cM window. The number of marker cofactors for background control was set by forward-backward stepwise regression with five controlling markers.

## Results

### Climate Conditions in the Two Locations for the Experimental Stage

**Figure 1:**
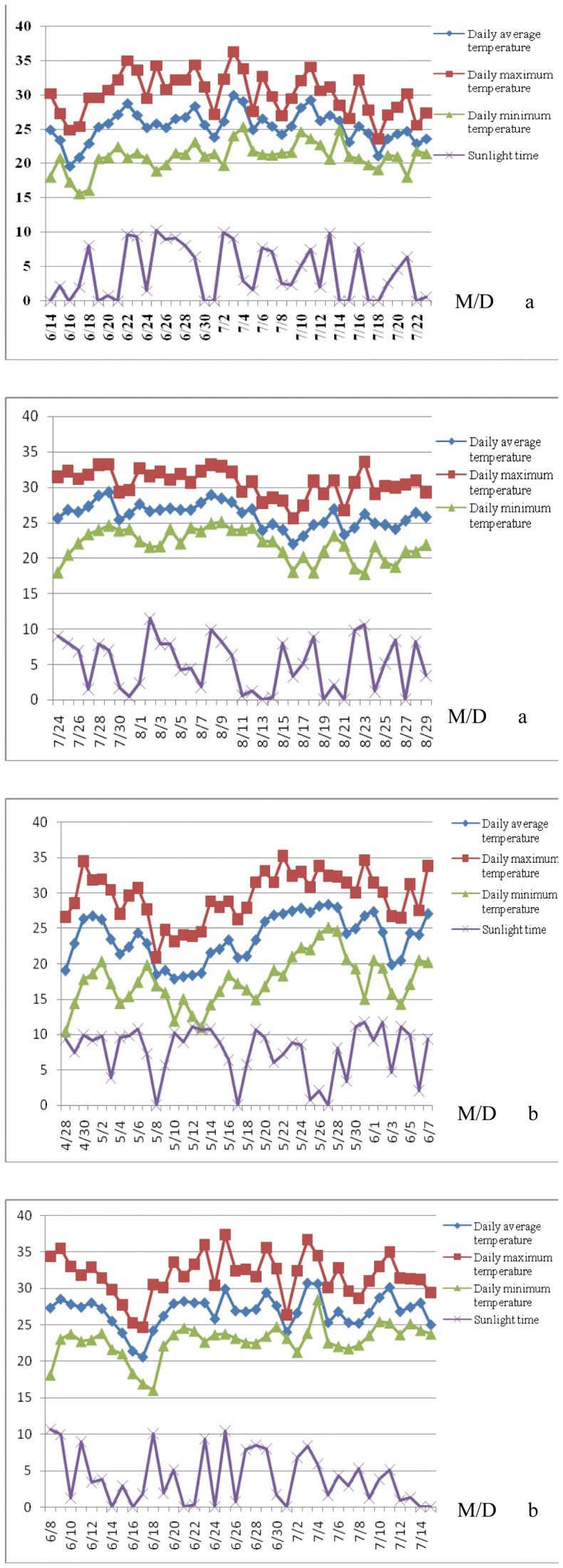
The four main climate factors in maize planting season and shading treatment season in 2008 at Xunxian (a) and Zhengzhou (b).

The amount of sunlight and temperature conditions during the experimental materials growing season and shading treatment at two locations were shown in Figure 1a and Figure 1b. The effective accumulated temperature and amount of sunlight from sowing date to shading treatment were 560.7°C, 313 hrs and 613.9°C, 166.7 hrs in Zhengzhou and Xunxian respectively, and the effective accumulated temperature and amount of sunlight between shading stage were 640.7°C, 154.4 hrs and 593.7°C, 183.2 hrs in Zhengzhou and Xunxian, respectively.

### The Performance of the Measured Traits under Shading Treatment

The six measured traits varied widely in the F_2:3_ populations under shading and full light (CK) treatment (Table 1). When grown under shading conditions, the plant height for the parent Zhong72 (P_1_) was reduced (relative to CK) by 12.66 cm (7.17%) and 17.13 cm (7.85%) at Zhengzhou and Xunxian, respectively (Table 1 and 2). The plant height for the other parent 502 (P_2_) was reduced by 35.20 cm (18.88%) and 31.46 cm (15.28%) at the same locations. For the hybrid, the plant height decreased by 8.20 cm (4.00%) and 21.26 cm (7.88%) under shading treatment comparing to CK at the two locations. The plant height of F_2:3_ populations were also lower under shading treatment at the two locations, and there was a wide variation under shading treatment comparing to CK.

**Table 1:** The performance of the six measured traits for shade tolerance in the F_2:3_ families under two treatments at two locations.

Location	Treatment	Trait ^a^	P_1_	P_2_	F_1_	F_2:3_ population	
						Range	Mean
Zhengzhou	Shade	PH (cm)	163.87	151.20	196.60	135.27–213.20	174.75±17.32
		EH (cm)	85.60	88.80	93.07	65.13–119.07	85.24±9.93
		SD (cm)	1.61	1.55	1.71	1.35–1.93	1.69±0.00
		DTT (d)	65.67	72.00	66.33	64.00–70.67	66.65±1.11
		DTS (d)	69.33	76.33	70.00	67.00–80.33	72.84±2.21
		ASI (d)	3.67	4.33	3.67	2.33–13.33	6.20±1.75
	CK	PH (cm)	176.53	186.40	204.80	155.60–235.73	193.92±16.1
		EH (cm)	75.27	90.93	91.07	64.13–109.67	91.27±8.66
		SD (cm)	1.76	1.98	2.04	1.69–2.41	2.03±0.13
		DTT (d)	64.67	66.67	62.67	61.33–69.33	64.91±1.37
		DTS (d)	67.33	68.67	66.33	63.67–75.33	69.50±2.41
		ASI (d)	2.67	2.00	3.67	1.33–8.67	4.59±1.47
Xunxian	Shade	PH (cm)	201.2	174.47	248.47	160.60–258.13	213.17±18.92
		EH (cm)	88.20	98.53	111.80	71.87–124.87	97.45±9.89
		SD (cm)	1.89	1.97	2.14	1.88–2.38	2.12±0.10
		DTT (d)	55.00	66.33	55.00	53.67–59.33	56.93±1.17
		DTS (d)	59.67	70.67	60.00	58.33–74.00	62.29±2.32
		ASI (d)	3.58	5.33	5.00	2.00–17.33	5.36±2.08
	CK	PH (cm)	218.33	205.93	269.73	173.87–280.73	227.50±18.01
		EH (cm)	90.27	106.07	119.07	75.33–124.4	98.12±9.30
		SD (cm)	1.92	2.13	2.27	1.94–2.63	2.27±0.12
		DTT (d)	53.00	61.33	51.33	51–57.67	54.75±1.54
		DTS (d)	57.33	65.00	57.00	53.33–64.00	59.64±1.56
		ASI (d)	2.67	3.67	5.67	1.33–9.00	4.89±1.32

Note:^ a^PH, plant height; EH, ear height; SD, stem diameter; DTT, day-to-tassel; DTS, day-to-silk; ASI, anthesis-silking interval.

b The broad-sense heritability of stover yield and its nutrient components.

c The confidence intervals of broad-sense heritability between 5% and 95% significant levels.

**Table 2:** The comparing value of the six measured traits for shading and full light treatment in the two parents, F_1_ and F_2:3_ populations at two locations.

Trait	Location	Zhengzhou				Xunxian			
	Population	P_1_	P_2_	F_1_	F_2:3_	P_1_	P_2_	F_1_	F_2:3_
PH	± CK	12.66	35.20	8.20	19.17	17.13	31.46	21.26	14.33
	%	7.17	18.88	4.00	9.89	7.85	15.28	7.88	6.30
SD	± CK	0.15	0.43	0.33	0.34	0.03	0.16	0.13	0.15
	%	8.52	21.72	16.18	16.75	1.56	7.51	5.73	6.61
EH	± CK	−10.33	2.13	−2.00	6.03	2.07	7.54	7.27	0.67
	%	−13.72	2.34	−2.20	6.61	2.29	7.07	6.11	0.68
DTT	± CK	−1.00	−5.33	−3.66	−1.74	−2.00	−5.00	−3.67	−2.18
	%	−1.55	−7.99	−5.84	−2.68	−3.77	−8.15	−7.15	−3.98
DTS	± CK	−2.00	−7.66	−3.67	−3.34	−2.34	−5.67	−3.00	−2.65
	%	−2.97	−11.15	−5.53	−4.81	−4.08	−8.72	−5.26	−4.44
ASI	± CK	−1.00	−2.33	0.00	−1.61	−0.91	−1.66	0.67	−0.47
	%	−37.45	−116.50	0.00	−35.08	−34.08	−45.23	11.82	−9.61

Ear height of the parent Zhong72 (P_1_) increased 10.33 cm (−13.72%) at Zhengzhou and decreased 2.07 cm (2.34%) at Xunxian under shading treatment compared to CK. Similarly, the ear height of the hybrid increased by 2.00 cm (−2.20%) and decreased by 7.27 cm (6.11%) at the same locations. However, ear height of the other parent, 502, decreased by 2.13 cm (23.4%) and 7.54 cm (7.07%) at Zhengzhou and Xunxian, respectively (Table 1 and 2). For the F_2:3_ populations, the average data of ear height were reduced at both locations.

For stem diameter, Zhong72 (P_1_) was reduced by 0.15 cm (8.52%) and 0.03 cm (1.56%) under shading treatment (relative to CK) at Zhengzhou and Xunxian locations, respectively. While the stem diameter of 502 (P_2_) was reduced by 0.43 cm (21.72%) and 0.16 cm (7.51%) at the same locations respectively. The hybrid was reduced by 0.33 cm (16.18%) and 0.13 cm (6.61%). In addition, the average value of the stem diameter in the F_2:3_ population under shading treatment was 1.69 cm at Zhengzhou location, with a 1.35–1.93 cm phenotypic variation, decreased 0.34 cm (16.75%). The average value of the stem diameter in the F_2:3_ population under full light treatment was 2.03 cm at the same location, with a 1.69–2.41 cm phenotypic variation. For the stem diameter of the F_2:3_ populations at Xunxian location, the average value was 2.12 cm under shading treatment comparing to 2.27 cm under full light treatment, with a 1.88 – 2.38 cm and 1.94 – 2.63 cm phenotypic variation, and decreased 0.15 cm (6.61%).

In parent Zhong72 DTT was delayed by 1 d (−1.55%) and 2 d (−3.77%) under shading treatment compared to CK at Zhengzhou and Xunxian, respectively. By contrast, the DTT in parent 502 was delayed by 5.33 d (−7.99%) and 5.00 d (−8.15%) at the same locations under shading treatment (relative to CK, Table 1 and 2), and the F_1_ delayed 3.66 d (−5.84%) and 3.67 d (−7.15%). In the F_2:3_ population, the average value of DTT was 66.65 d (range; 64.00–70.67 d) under shading treatment at Zhengzhou. However, the average DTT was 64.91 d with a range of 61.33–69.33 d under full light treatment, and DTT was also reduced under shading treatment at Xunxian. Additionally, DTS showed similar results for the experimental materials under shading and full light treatment.

The ASI in the parent Zhong72 increased by 1 d (−37.45%) and 0.91 d (−34.08%) under shading treatment compared to CK at Zhengzhou and Xunxian, respectively. In parent 502, ASI increased by 2.33 d (−116.50%) and 1.66 d (−45.23%) under shading treatment (relative to CK) at the same locations. However, the ASI of the hybrid did not change at Zhengzhou, but decreased by 0.67 d at Xunxian. In the F_2:3_ populations, the average ASI was 6.20 d under shading treatment at Zhengzhou, with a 2.33–13.33 d phenotypic variation, compared with average data of 4.59 d with a 1.33–8.67 d variation under natural sunlight.

**Figure 2:**
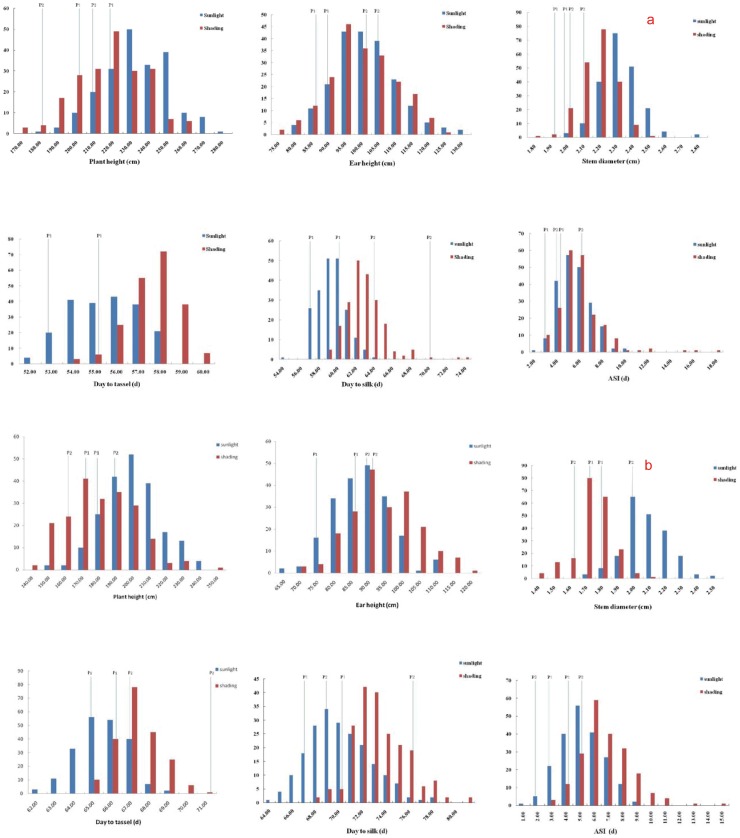
Histogram of the six measured traits in the F2:3 populations at Xunxian (a) and Zhengzhou (b) location.

Totally, Comparing to full sunlight, plant height and stem diameter of the two parents, F_1_ and F_2:3_ populations all decreased at shading treatment at two locations, on the contrary, DTT, DTS and ASI increased at the shading treatment at two locations simultaneously (Figure 2a and 2b, Table 2). The results demonstrated that shading treatment at middle and late growing stage in maize could decrease plant height, shortened stem diameter, extended tassel and silk times, and increased anthesis-silking time. However, the ear height of the F_2:3_ populations under shading treatment were significantly increase at Zhengzhou location and a little decrease at Xunxian location, this result implied that the variation of ear height was not only effect by shading treatment but also could be effect by other meteorological factors such as effective accumulated temperature because of the effective accumulated temperature and amount of sunlight at two locations (Figure 1a and 1b; Figure 2a and 2b).

There were significant differences for the six measured treats in the F_2:3_ populations between the two treatments and genotypes, as well as the interaction of shading and locations (p<0.01, Table 3). However, no significant differences were noted for ASI and SD in the F_2:3_ populations from different locations. In the six trait related to shading sensitivity, plant height and ear height had high broad-sense heritability (91.4% and 91.9%), then were stem diameter (82.4%), day to tassel (80.8%) and day to silk (80.3%), the least was stem diameter (64.4%).

**Table 3:** Variance analysis of the six measured traits for shading and full light treatment in the F_2:3_ populations at two locations.

Source of variance	DTT	DTS	ASI	SD	PH	EH
L	61922.43^**^	65061.83^**^	38.81	70.54	815259.39^**^	57819.37
B	12.13^**^	30.44^**^	13.23^**^	1.41^**^	6279.64^**^	305.78^**^
G	15.21^**^	39.77^**^	22.87^**^	0.09^**^	3132.50^**^	887.60^**^
S×L	28.64^**^	77.90^**^	200.99^**^	5.75^**^	3457.75^**^	7119.33^**^
S×G	2.27	6.76*	4.78^**^	0.03	206.42^**^	54.98
S×L×G	2.03^**^	5.09^**^	3.09^**^	0.02	145.49	50.03

Note: L, location; B, block; G, genotype; S, shading treatment.

* ,**: the significant at the 0.05 and 0.01 levels.

### Construction of Genetic Linkage Maps

**Figure 3:**
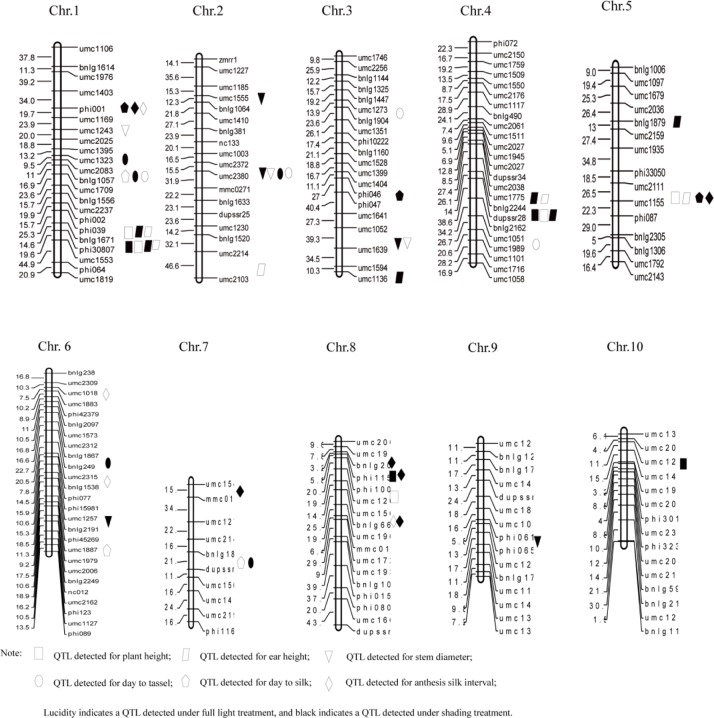
Chromosomal location of quantitative trait loci (QTL) for shading sensitive related traits in maize under two shading treatments. The genetic distance in cM is listed on the left side of each chromosome.

In the 210 SSR markers possessed distinct polymorphisms were chosen to amplify the F_2_ population DNA, only 197 polymorphic SSR markers could link on 10 chromosomes according to linkage analysis by using the Mapmakers 3.0 at a LOD threshold >3.0. Based on the genotypes of the molecular markers, they covered 10 chromosomes, spanned 2693.6 cM, and had a 13.67 cM average interval between markers (Figure 3). These characteristics were consistent with linkage maps published in the maize genome database (www.maizegdb.org).

### QTL Analysis for the Shading Sensitive Traits

A total of 43 different QTL were identified for the six measured traits under shading and full light treatment at the two locations, and the QTL were distributed over all 10 chromosomes (Table 4, Figure 3). There were seven different QTL detected for plant height under shading and full sunlight treatment at two locations. Of these, QTL *qPH4*, was identified at all treatments and locations simultaneously, and contributed 9.72%, 13.31%, 8.61%, and 9.12% of the phenotypic variation of plant height under shading treatment and full-light treatment at Xunxian and Zhengzhou, respectively, which could increase 5.19 cm, 5.93 cm, 9.99 cm and 7.60 cm to plant height. Another QTL, *qPH8b*, was detected only under full light treatment (CK) at two locations simultaneously. The *qPH8b* explained 14.16% and 12.38% of the phenotypic variation in plant height at Xunxian and Zhengzhou, respectively. Both of these QTL were derived from the parent Zhong72. Another QTL, *qPH10*, was detected only under shading treatment at the two locations, and explained 10.48% and 8.74% of the phenotypic variation for plant height at Zhengzhou and Xunxian, respectively. The *qPH10* allele was derived from the parent 502.

**Table 4:** QTL detected for six measured traits related to shading under two treatments at two locations.

Location	Treatment	Trait	QTL	Flanking-marker	A^a^	D^a^	D/A^b^	Gene action^b^	R^2c^
Xunxian	Shade	PH	*qPH4*	dupssr28-bnlg2162	5.19	5.10	0.98	D	9.72
			*qPH8a*	phi0017-umc1202	5.73	2.97	0.52	PD	12.13
			*qPH10*	umc1432-umc1962	−10.48	7.29	−0.70	PD	10.10
		EH	*qEH1a*	phi039-bnlg1671	−2.83	−5.54	1.96	OD	15.25
			*qEH4a*	dupssr28-bnlg2162	2.06	2.66	1.29	OD	7.59
		SD	*qSD2b*	umc2372-umc2380	−0.05	−0.01	0.25	PD	8.05
			*qSD6*	umc2006-bnlg2249	−0.07	0.03	−0.48	PD	11.96
			*qSD9*	phi065-umc1271	−0.05	0.01	−0.29	PD	7.82
		DTS	*qDTS1a*	umc1403-phi001	−0.85	−0.38	0.45	PD	10.02
			*qDTS5*	umc2111-umc1155	−0.70	−0.68	0.98	D	20.62
		DTT	*qDTT1b*	umc2083-bnlg1057	−0.45	0.07	−0.16	A	8.43
			*qDTT2*	umc2372-umc2380	−0.76	0.19	−0.25	PD	9.38
			*qDTT6*	phi15981-umc1257	0.52	−0.12	−0.23	PD	7.72
			*qDTT7*	bnlg1808-dupssr9	0.58	−0.77	−1.33	OD	11.62
		ASI	*qASI1*	umc1403-phi001	−0.72	−0.30	0.42	PD	9.36
			*qASI5*	umc2111-umc1155	−0.51	−0.58	1.13	D	22.39
			*qASI8a*	phi10017-umc1202	−0.32	0.04	−0.13	A	33.30
			*qASI8b*	umc1202-umc1562	−0.18	−0.21	1.13	D	32.87
	CK	PH	*qPH1a*	phi039-bnlg1671	−1.82	−12.09	6.63	OD	15.03
			*qPH1b*	bnlg1671-phi30807	−1.26	−11.99	9.50	OD	12.15
			*qPH4*	dupssr28-bnlg2162	5.93	5.54	0.93	D	13.31
			*qPH8b*	umc1562-bnlg666	3.03	4.90	1.62	OD	14.16
		EH	*qEH1a*	phi039-bnlg1671	−3.06	−5.04	1.65	OD	16.74
			*qEH1b*	bnlg1671-phi30807	−3.16	−6.11	1.93	OD	18.13
			*qEH4a*	dupssr28-bnlg2162	2.32	1.12	0.48	PD	13.23
			*qEH5a*	umc2111-umc1155	−3.72	−0.98	0.26	PD	11.12
		SD	*qSD1*	umc1169-umc1243	−0.04	0.00	−0.02	A	21.25
			*qSD2b*	umc2372-umc2380	−0.07	−0.01	0.10	A	9.89
		DTS	*qDTS1b*	umc2083-bnlg1057	−0.82	0.31	−0.38	PD	11.53
			*qDTS7*	bnlg1808-dupssr9	1.02	−0.67	−0.66	PD	10.04
		DTT	*qDTT1b*	umc2083-bnlg1057	−0.85	0.26	−0.31	PD	14.39
			*qDTT2*	umc2372-umc2380	−0.88	−0.25	0.28	PD	13.76
			*qDTT3*	umc1273-bnlg1904	0.71	−0.08	−0.12	A	8.87
		ASI	*qASI6a*	umc1018-umc1883	−0.97	0.16	−0.17	A	12.77
			*qASI8c*	mmc0181-umc1724	0.42	0.20	0.48	PD	8.77
Zhengzhou	Shade	PH	*qPH1b*	bnlg1671-phi30807	−3.62	−9.42	2.60	OD	9.39
			*qPH4*	dupssr28-bnlg2162	9.99	−2.71	−0.27	PD	8.61
			*qPH10*	umc1432-umc1962	−8.74	3.29	−0.38	PD	7.90
		EH	*qEH1a*	phi039-bnlg1671	−1.25	−5.94	4.76	OD	14.23
			*qEH1b*	bnlg1671-phi30807	−2.04	−5.58	2.74	OD	12.70
			*qEH3*	umc1594-umc1136	5.21	−2.02	−0.39	PD	7.52
			*qEH4a*	dupssr28-bnlg2162	3.47	1.03	0.30	PD	9.40
			*qEH4b*	bnlg2162-umc1051	4.26	0.29	0.07	A	8.78
			*qEH5b*	umc2036-bnlg1879	−4.14	0.29	−0.07	A	11.26
		SD	*qSD2a*	umc1185-umc1155	−0.07	0.02	−0.27	PD	7.86
			*qSD3*	umc1052-umc1639	−0.02	0.07	−3.63	OD	9.53
		DTS	*qDTS1a*	umc1403-phi001	−0.39	−1.23	3.12	OD	14.51
			*qDTS3*	phi046-phi047	1.32	−0.47	−0.35	PD	12.32
		DTT	*qDTT1a*	umc1395-umc1323	−0.40	−0.19	0.48	PD	11.95
			*qDTT1b*	umc2083-bnlg1057	−0.49	0.07	−0.15	A	15.97
		ASI	*qASI7*	umc1546-mmc0171	0.84	−0.06	−0.07	A	9.69
			*qASI8c*	mmc0181-umc1724	0.49	0.26	0.52	PD	10.26
	CK	PH	*qPH4*	dupssr28-bnlg2162	7.60	0.27	0.04	A	9.12
			*qPH5*	umc2111-umc1155	−7.49	−2.19	0.29	PD	15.07
			*qPH8b*	umc1562-bnlg666	4.56	2.28	0.50	PD	12.38
		EH	*qEH2*	umc2214-umc2103	−4.67	2.84	−0.61	PD	7.54
			*qEH4a*	dupssr28-bnlg2162	3.72	−1.10	−0.30	PD	5.97
			*qEH5a*	umc2111-umc1155	−3.31	−2.16	0.65	PD	11.49
		SD	*qSD3*	umc1052-umc1639	0.02	0.06	2.56	OD	9.14
		DTS	*qDTS1b*	umc2083-bnlg1057	−1.25	0.64	−0.51	PD	8.68
			*qDTS6*	umc1127-phi089	0.20	1.25	6.29	OD	9.43
		DTT	*qDTT1b*	umc2083-bnlg1057	−0.66	0.43	−0.65	PD	8.99
			*qDTT2*	umc2372-umc2380	−0.55	−0.46	0.84	D	13.94
			*qDTT4*	umc1989-umc1101	−0.41	−0.25	0.61	PD	7.81
		ASI	*qASI1*	umc1403-phi001	−0.55	−0.31	0.57	PD	11.38
			*qASI6b*	phi45269-umc1887	−0.88	0.72	−0.81	D	8.14

Note: ^a^Additive effect; positive values of the additive effect indicate that the Zhong72 alleles are in the direction of increasing the traits. Dominance effect; positive values of the dominance effect indicate that the heterozygotes have higher phenotypic values than the respective means of the two homozygotes, and negative values indicate that heterozygotes have lower values than the means of the two homozygotes.

b A, additive (d/a = 0.00–0.20); PD, partial dominance (d/a = 0.21–0.80); D, dominance (d/a = 0.81–1.20); OD, overdominance (d/a>1.20).

c R^2^ contribution rate.

Nine different QTL for ear height were identified in this study. At Zhengzhou, two and four QTL were found under shading and full light treatment, respectively. In contrast, six and two QTL were identified for shading and full light treatment at Xunxian. The QTL *qEH4a* was detected under both treatments at both locations simultaneously, and explained 7.59%, 8.21%, 9.40%, and 5.97% of the phenotypic variation in ear height under shading and full light treatment at Xunxian and Zhengzhou, respectively, and the *qEH4a* allele was derived from the parent Zhong72. Another QTL, *qEH1a*, was identified under shading and full sunlight treatments at Zhengzhou and shading treatment at Xunxian, explaining 15.25%, 16.74%, and 14.23% of the variation in ear height. In addition, QTL *qEH5a* was detected under full light treatment at two locations, explaining 11.12% and 11.49% of the phenotypic variation in ear height at Xunxian and Zhengzhou, respectively. The cumulative contribution ratio (R^2^) detected for ear height explained 22.84% and 54.09% of the phenotypic variance under shading and full light treatment, respectively, at Zhengzhou. In contrast, 63.89% and 24.63% of the phenotypic variance was explained under shading and full light treatment at Xunxian, respectively.

Three and two QTL were identified for stem diameter (SD) under shading treatment at Zhengzhou and Xunxian, respectively, and two and one QTL were detected under full light treatment at Zhengzhou and Xunxian, respectively. The QTL *qSD2b* was detected under shading and full sunlight treatment at Zhengzhou simultaneously. The allele was derived from 502 and was associated with increased stem diameter. The QTL *qSD3* was detected under both treatments at Xunxian, resulting in a 9.53% and 9.14% phenotypic variance of stem diameter under shading and full light treatment. The effects resulted from the allele of parent Zhong72.

For DTT, there were four and three QTL revealed under shading and full light treatment, respectively, at Xunxian, while two and three QTL were detected under the same treatments at Zhengzhou respectively. A total of seven different QTL were revealed. The QTL *qDTT1b* explained 8.43%, 14.39%, 11.95%, and 8.99% of the phenotypic variance of DTT under shading and full light treatment at Xunxian and Zhengzhou, respectively. The effects resulted from the allele derived from the parent 502. Another QTL, *qDTT2*, was detected under both treatments at Xunxian and under full light treatment at Zhengzhou, and explained 9.38%, 13.76%, and13.94%, respectively, of the DTT phenotypic variance.

Six different QTL were identified for DTS under the two treatments at both locations. QTL *qDTS1a* was detected under shading treatment at two locations, explaining 10.02% and 14.51% of the DTS phenotypic variance at Xunxian and Zhengzhou, respectively. This QTL resulted from the direct effects of the allele from the parent 502. Another QTL, *qDTS1b*, was detected under full light treatment at two locations, explaining 11.53% and 8.68% of the DTS phenotypic variance (from Xunxian and Zhengzhou, respectively). The allele derived from 502 was associated with increased DTS.

Eight different QTL were found to be associated with ASI under shading treatment at both locations. Four QTL were also detected under full light treatment. QTL *qASI1*, derived from the parent 502, contributed 9.36% and 11.38% of the ASI phenotypic variance under shading treatment at Xunxian and full light treatment at Zhengzhou, respectively. Another QTL, *qASI8c*, was detected under full light treatment at Xunxian and shading treatment at Zhengzhou, contributing 8.77% and 10.26% to the ASI phenotypic variance, respectively, and the alleles were derived from the parent Zhong72.

## Discussion

Many reports have shown that shading stress is an important abiotic factor that reduces grain yield during maize development and reproductive stage. Early et al. found that vegetative growth and kernel number were greatly reduced relative to controls when grown under more extreme shading (80–90% interception of incident light) treatment during vegetative development [2]. When plants were shaded during flowering, photosynthesis decreased, and kernel abortion increased relative to controls [1]. Reduction of incident light, particularly during reproductive growth, causes a sever reduction in grain yield, mainly through a decrease in kernel number [5]. Thus, photosynthate supply has a substantial impact on kernel set, as indicated by studies involving altered illumination levels [32]–[34]. In the present study, we found that the six measured traits were significantly influenced by shading treatment compared to full light during the plant development and reproductive stages (Table 1 and 2), and shading treatment could decrease plant height, reduce stem diameter, delay DTT and DTS, and increase ASI (Figure 2a and 2b). These results are consistent with previous studies [14], [35].

However, an interesting phenomenon had been found in this report, ear height of the parent Zhong72 had opposite result at two locations under shading treatment compared to full sunlight treatment. Tang et al. have reported that the number of internode and ear inserted internode was always stable at different environments for an inbred line or hybrid, plant height and ear height was mainly decided by the elongation of internodes [36]. Fournier and Andrieu have report that internode length was determined as a function of thermal time by measuring the vertical displacement of individual leaf collars, and the onset of the linear phase of elongation for internodes was delayed by shading, but its duration was not affected when shading was applied after the tip appearance of leaf 6. The reduction in the linear elongation rate was almost totally responsible for the reduction in the final length of phytomers in the shade treatment [8]. In this study, the experimental materials were planted at two locations at different seasons, the effective accumulated temperature, especially the amount of sunlight from sowing to shading treatment were significant different. Owing to ear height was easy affected by early developing stages because of the elongation of lower internodes, and the inbred line Zhong72 had a little photoperiod sensitive, so it showed an opposite performance at shading treatment comparing to sunlight treatment in this study.

When comparing to the different value of the six measured traits for the two parents and F_1_ under shading and full sunlight treatment, the percent of the increasing value of DTT and DTS for F_1_ were between two parents at two locations simultaneously, so was the percent of decreasing value for stem diameter (Table 2). However, the percent of the decreasing value of plant height and the increasing value of anthesis-silking interval were beyond the low value parent (Zhong72) and high value parent (502) at two locations, respectively. This result showed that F_1_ had over-parent heterosis of the increasing or decreasing percent for anthesis-silking interval and plant height, and hybrid had strong suffertibility than the two inbred lines for the two traits under shading treatment.

Out of the six measured traits in this study, five agricultural traits including to plant height, ear height, day to tassel, day to silk and anthesis-silking interval has been reported the QTL mapping results in previous studies (Table 4). Comparing the chromosomal region of the QTL detected in this paper and previous reports, in the chromosomal region of the QTL *qPH1a*, five QTL including *qplht20*, *qplht71*, *qplht75*, *qplht85*, *qplht168* have been found in previous studies [37]–[41]. The most widely reported QTL were *qPH8a* (the pervious indentified QTL *qplhtws3*, *qplht25*, *qplht26*, *qplht39*, *qplht51* and *qplht156)* [36], [42]–[45] and *qPH4* (the corresponding QTL, *qplht67*, *qplht73* and *qplht145*) [37], [45]. The other QTL *qPH1b*, *qPH5*, *qPH8b* and *qPH10* also have been identified as *qplht136*, *qplht149*, *qplht45* and *qplht161* in the same chromosomal regions [45]–[47]. For ear height, the chromosomal region of the QTL *qEH1a* has been detected for four QTL (*qearhl1*, *qearhl6*, *qearhl9* and *qearhl22*) for ear height [39]–[40], and the others QTL, *qEH1b, qEH4a* and *qEH5a* also have been identified as *qearhl39*, *qearht25* and *qearht13* [39]–[40], [48]. In the chromosomal region of *qDTT1b* detected in this study, six QTL (*qdpoll10*, *qdpoll20*, *qdpoll26*, *qdpoll32*, *qdpoll39* and *qdpoll45*) have been identified for day to tassel [44], [49], and for the chromosomal regions of the QTL *qDTT2* and *qDTT3* detected in this study, three and two QTL (*qdpoll29*, *qdpoll40*, *qdpoll46* and *qdpoll7*, *qdpoll55*) for day to pollen have been identified [40]–[41], [49]–[50]. Three and three QTL for day to silk (*qdsilk12*, *qdsilk18*, *qdsilk23* and *qdsilk13*, *qdsilk19*, *qdsilk45*) identified by previous studies [49], [51] were situated at the same chromosomal loci as the QTL *qDTS1a* and *qDTS1b* reported in this study, and another research have reported the QTL *qDTS3* and *qDTS5* [52]. For anthesis-silking interval, five QTL *qASI1*, *qASI6b*, *qASI8a*, *qASI8b* and *qASI8c* detected in this study have the same chromosomal regions as the QTL *qasi45*, *qasi33*, *qasi28*, *qasi42*, *qasi34* and *qasi48* identified in previous studies [14], [42], [49]. These comparisons substantiate the existence of QTL and show that QTL can be identified by crossing different background material in different environments.

In the grasses, light quality and intensity have a profound effect on the developmental progression of vegetative meristem development [53]. Artificial shading may have effects similar to those of high densities. Hashemi-Dezfouli and Herbert reported that the rate of apparent photosynthesis in ear leaves was reduced significantly by both increased plant density and shading [34]. Tassel emergence was slightly delayed in high density and shaded plots. Gerakis and Papkosta-Tasopoulou showed that yield reduction per plant, brought about by 50% artificial shading, was approximately equivalent to increasing plant density from 5 to 12.5 plants m^−2^ [7]. Other investigators have reported similar associations between tolerance of hybrids to artificial shading and tolerance to self-shading under high plant density conditions [7], [14]. The reduction was attributed to reduce photosynthetically active radiation (PAR) in higher densities and shaded plots, and to the decreased chlorophyll concentration measured in leaves of plants at grown at high density in both ambient light and shaded plots [32]. However, in many countries, increasing plant density has been become an important tactic for obtaining high grain yield [54]. However, increasing plant density has been proved to have a similar influence as shading treatment in the field [7], [14]. Thus, selecting elite hybrids with strong tolerance to high density and shading stress has become an important target in maize breeding, especially in the middle latitudes, with their relatively short developing period for maize. In this study, we found that the inbred line 502 was more sensitive than Zhong72 to shading treatment as previous appraised result, and the inbred line Zhong72 can be used as a good germplasm for selecting insensitive inbred line in maize breeding. Additionally two important QTL *qPH4* and *qEH4a* for plant height and ear height, which derived from the parent Zhong72, and the *qDTT1b* for DTT coming from the parent 502, were detected under shading and full light treatment at two locations simultaneously, these QTL had insensitive for shading stress and could be used to select elite hybrids with strong tolerance for shading stress and/or high plant density in a maize breeding procedure. On the contrary, the two QTL (*qPH10* and *qDTS1a*) for plant height and DTS were detected under shading treatment at two locations only, which had sensitive to shading stress, and should be quit in maize breeding procedure by means of MAS method especially in the middle latitudes.

## References

[1] Reed AJ, Singletary GW, Schussler JR, Williamson DR, Christy AL (1988). Shading effect on dry matter and nitrogen partitioning, kernel number, and yield of maize. Crop Sci..

[2] Early EB, McIlrath WO, Seif RD, Hageman RH (1967). Effects of shade applied at different stages of plant development on corn (*Zea mays* L.) production. Crop Sci..

[3] Schmidt WH, Colville WL (1967). Yield and yield components of *Zea mays* L. as influenced by artificial shading. Crop Sci..

[4] Tollenaar M (1977). Sink-source relationships during reproductive development in maize. A review.. Maydica.

[5] Kiniry JR, Ritchie JT (1985). Shade-sensitive interval of kernel number of maize. Agron. J..

[6] Tollenaar M, Daynard TB (1978). Relationship between assimilate source and sink in maize in a short-season environment. Agron. J..

[7] Gerakis PA, Papakosta-Tasopoulou D (1980). Effects of dense planting and artificial shading on five maize hybrids. Agric. Meteorol..

[8] Fournier C, Andrieu B (2000). Dynamics of the elongation of internodes in maize (*Zea mays* L.). Effects of shade treatment on elongation patterns.. Annals of Botany.

[9] Struik PC (1983). The effects of short and long shading, applied during different stages of growth, on the development, productivity, and quality of forage maize (*Zea mays* L.). Neth. J. Agric. Sci..

[10] Stinson HT, Moss DN (1960). Some effects of shade upon corn hybrids tolerant and intolerant of dense planting. Agron. J..

[11] Setter TL, Flannigan BA, Melkonian J (2001). Loss of kernel set due to water deficit and shade in maize: carbohydrate supplies, abscisic acid, and cytokinins. Crop Sci..

[12] Edmeades GO, Bolanos J, Elings A, Ribaut JM, Banziger JM (2000). The role and regulation of the anthesis-silking interval in maize. In Westgate M E and Boote K J (ed.) Physiology and modeling kernel set in maize.. CSSA, Madison, WI, pp 43–73.

[13] Ward DA, Woolhouse HW (1986). Comparative effects of light during growth on the photosynthetic properties of NADP-ME type C_4_ grasses from open and shaded habitats. I. Gas exchange, 1eaf anatomy and ultrastructure.. Plant, Cell and Environment.

[14] Moss DN, Stinson HT (1961). Differential response of corn hybrids to shade. Crop Sci..

[15] Schoper JB, Johnson RR, Lambert RJ (1982). Maize yield response to increased assimilate supply. Crop Sci..

[16] Karlen DL, Camp CR (1985). Row spacing, plant population, and water management effects on corn in the Atlantic Coastal Plain. Agron. J..

[17] Early EB, Miller RJ, Hageman RH, Seif RD (1966). Effects of shade on maize production under field conditions. Crop Sci..

[18] Hébert Y, Guingo E, Loudet O (2001). The response of root/shoot partitioning and root morphology to light reduction in maize genotype. Crop Sci..

[19] Liu WD, Tollenaar M (2009). Physiological mechanisms underlying heterosis for shade tolerance in maize. Crop Sci..

[20] Agrama HA, Moussa ME (1996). Mapping QTLs in breeding for drought tolerance in maize (Zea mays L.).. Euphytica.

[21] Ribaut J, Hoisington D, Deutsch JA, Jiang CZ, Gonzalez de Leon D (1996). Identification of quantitative trait loci under drought conditions in tropical maize. 2. Flowering parameters and the anthesis-silking interval.. Theor Appl Genet.

[22] Veldboom L, Lee M (1996). Genetic mapping of quantitative trait loci in maize in stress and nonstress environments. II. Plant height and flowering.. Crop Sci.

[23] Khairallah M, Bohn M, Jiang CZ, Deutsch JA, Jewell DC (1998). Molecular mapping of QTL for southwestern corn borer resistance, plant height and flowering in tropical maize.. Z Pflanzenzuecht.

[24] Welcker C, Boussuge B, Bencivenni C, Ribaut JM, Tardieu F (2007). Are source and sink strengths genetically linked in maize plants subjected to water deficit? A QTL study of the responses of leaf growth and of Anthesis-Silking Interval to water deficit.. Journal of Experimental Botany.

[25] Marino R, Ponnaiah M, Krajewski P, Frova C, Gianfranceschi L (2009). Addressing drought tolerance in maize by transcriptional profiling and mapping.. Mol Genet Genomics.

[26] Messmer R, Fracheboud Y, Bänziger M, Vargas M, Stamp P (2009). Drought stress and tropical maize: QTL-by-environment interactions and stability of QTLs across environments for yield components and secondary traits.. Theor Appl Genet.

[27] Yuan LZ, Li CH, Wang XP, Yang SK (2008). Comparison of shade tolerance among different maize (*Zea Mays* L.) inbred Line.. J. of Maize Science.

[28] Ben Haj Salah H, Tardieu F (1996). Quantitative analysis of the combined effects of temperature, evaporative demand and light on leaf elongation rate in well-watered field and laboratory-grown maize plants.. Journal of Experimental Botany.

[29] Knapp SJ, Stroup WW, Ross WM (1985). Exact confidence intervals for heritability on a progeny mean basis. Crop Sci..

[30] Lander ES, Green P, Abrahamson J, Barlow A, Daly MJ (1987). MAPMAKER: an interactive computer package for constructing primary genetic linkage maps of experimental and natural populations.. Genomics.

[31] Zeng ZB (1994). Precision mapping of quantitative trait loci.. Genetics.

[32] Andrade FH, Vega C, Uhart S, Cirilo A, Cantarero M (1999). Kernel number determination in maize. Crop Sci..

[33] Setter TL, Flannigan BA (1989). Relationship between photosynthate supply and endosperm development in maize. Ann. Bot..

[34] Singh BK, Jenner CF (1984). Factors controlling endosperm cell number and grain dry weight in wheat: effects of shading on intact plants and of variation in nutritional supply to detached cultured ears. Aust. J. Plant Physiol..

[35] Hashemi-Dezfouli A, Herbert SJ (1992). Intensifying plant density response of corn with artificial shade.. Agronomy Journal.

[36] Tang JH, Teng WT, Ma XQ, Yan JB, Meng YJ (2007). The genetic dissection of plant height using a set of RIL population in maize.. Euphytica.

[37] Beavis WD, Grant D, Albertsen MC, Fincher RR (1991). Quantitative trait loci for plant height in four maize populations and their associations with qualitative genetic loci.. Theor Appl Genet.

[38] Schon CC, Melchinger AE, Boppenmaier J, Brunklaus-Jung E, Herrmann RG (1994). RFLP mapping in maize - quantitative trait loci affecting testcross performance of elite European flint lines.. Crop Sci.

[39] Veldboom LR, Lee M, Woodman WL (1994). Molecular marker-facilitated studies in an elite maize population: 1. Linkage analysis and determination of QTL for morphological traits.. Theor Appl Genet.

[40] Veldboom LR, Lee M (1996). Genetic mapping of quantitative trait loci in maize in stress and nonstress environments. 2. Plant height and flowering.. Crop Sci.

[41] Khairallah M, Bohn M, Deutsch JA, Jewell DC, Mihm JA (1998). Molecular mapping of QTL for southwestern corn borer resistance, plant height and flowering in tropical maize.. Z Pflanzenzuecht.

[42] Messmer R, Fracheboud Y, Banziger M, Vargas M, Stamp P (2009). Drought stress and tropical maize: QTL-by-environment interactions and stability of QTLs across environments for yield components and secondary traits.. Theor Appl Genet.

[43] Beavis WD, Smith OS, Grant D, Fincher R (1994). Identification of quantitative trait loci using a small sample of topcrossed and F4 progeny from maize.. Crop Sci.

[44] Koester RP, Sisco PH, Stuber CW (1993). Identification of quantitative trait loci controlling days to flowering and plant height in two near isogenic lines of maize.. Crop Sci.

[45] Lübberstedt T, Melchinger AE, Schoen CC, Utz H, Klein D (1997). QTL mapping in testcrosses of European flint lines of maize. 1. Comparison of different testers for forage yield traits.. Crop Sci.

[46] Melchinger AE, Utz H, Schoen CC (1998). Quantitative trait locus (QTL) mapping using different testers and independent population samples in maize reveals low power of QTL detection and large bias in estimates of QTL effects.. Genetics.

[47] Koester RP, Sisco PH, Stuber CW (1993). Identification of quantitative trait loci controlling days to flowering and plant height in two near isogenic lines of maize.. Crop Sci.

[48] Berke T, Rocheford T (1995). Quantitative trait loci for flowering, plant and ear height, and kernel traits in maize.. Crop Sci.

[49] Ribaut J, Hoisington D, Deutsch JA, Jiang CZ, Gonzalez de Leon D (1996). Identification of quantitative trait loci under drought conditions in tropical maize. 2. Flowering parameters and the anthesis-silking interval.. Theor Appl Genet.

[50] Kozumplik V, Pejic I, Senior L, Pavlina R, Graham GI (1996). Molecular markers for QTL detection in segregating maize populations derived from exotic germplasm.. Maydica.

[51] Rebai A, Blanchard P, Perret D, Vincourt P (1997). Mapping quantitative trait loci controlling silking date in a diallel cross among four lines of maize.. Theor Appl Genet.

[52] Abler BS, Edwards M, Stuber CW (1991). Isoenzymatic identification of quantitative trait loci in crosses of elite maize inbreds.. Crop Sci.

[53] Doust AN (2007). Grass architecture: genetic and environmental control of branching.. Current Opinion in Plant Biology.

[54] Duvick DN (1999). Heterosis: feeding people and protecting natural resources.. In: Coors J G, Pandey S (eds) Genetics and exploitation of heterosis in crops. Am Soc Agron, Crop Sci Soc Am, Soil Sci Soc Am, Inc, Madison, Wisconsin, USA, pp 19–29..

